# Aluminum

**Published:** 1891-09

**Authors:** 


### ALUMINUM.

Aluminum is a white, ductile, metallic substance, resembling silver. It is susceptible of a higher polish than silver, and is not affected by atmospheric changes. Sulphur, nitric acid and diluted sulphuric acid do not injure it in the least. A solution of caustic potash or soda, however, will dissolve the metal with great ease. It is also readily soluble in dilute hydrochloric acid, with evolution of hydrogen.

This peculiar metal was first discovered in 1828, by Wohler, who obtained it from chloride. Thirty years later the mode of production was so simplified by Deville that it could be produced in sufficient quantities for manufacturing purposes. The process was, neverthe-less, too expensive to warrant a very extensive use of the new metal, the price, until recently, being as high as $13.00 a pound. By an improved process of electrolysis, fully protected by letters patent, aluminum is now obtained from clay, so easily, that it has been sold in any quantity desired for from $1.50 to $2.00 a pound. Recently competition has reduced the price to a dollar a pound, and from present indication, we are warranted in stating that the price will still see a remarkable reduction.

The new metal is extremely malleable and ductile, and may be rolled into thin sheets, or drawn into fine wire. By cold hammering it becomes hard as soft iron, but by fusion may be softened again. It is very light, being only two and one-half times heavier than water; the weight of a given bulk of Aluminum being 1, iron is 2.9 times as heavy; copper, 3.6 times as heavy; nickel, 3.5 times as heavy; silver, 4 times as heavy; lead, 4.8 times as heavy; gold, 7.7 times as heavy; and platinum 9 times as heavy.

With the cheapening of manufacture this valuable metal is coming rapidly to the front, and will, no doubt, revolutionize the mechanical world. It is already used for ornaments, jewelry, medals, cutlery, culinary articles, pens, match boxes, thimbles, etc., etc.

